# Supplementary material for: Climate variability on Fit for 55 European power systems
Source: PLoS One. 2023 Dec 20;18(12):e0289705. doi: 10.1371/journal.pone.0289705 (PMC10732370; doi:10.1371/journal.pone.0289705)

# Climate variability on Fit for 55 European power systems

## Supplementary materials

Figs S1 and S2 show how the generation from RES sources varies for each region (Fig S1) or each source (Fig S2) and for each week of the year. For hydropower, we use the inflow rather than the generation, in order to focus on the natural variability of the sources, excluding the impact of electricity dispatching (most of the hydropower in Europe is dispatchable).

The inter-annual variability of solar, wind and inflow is shown in Figs S3-S5.

**Fig S1**. Weekly average RES production for each region in TWh. The colour represents the coefficient of variation for each week considering all the climate years


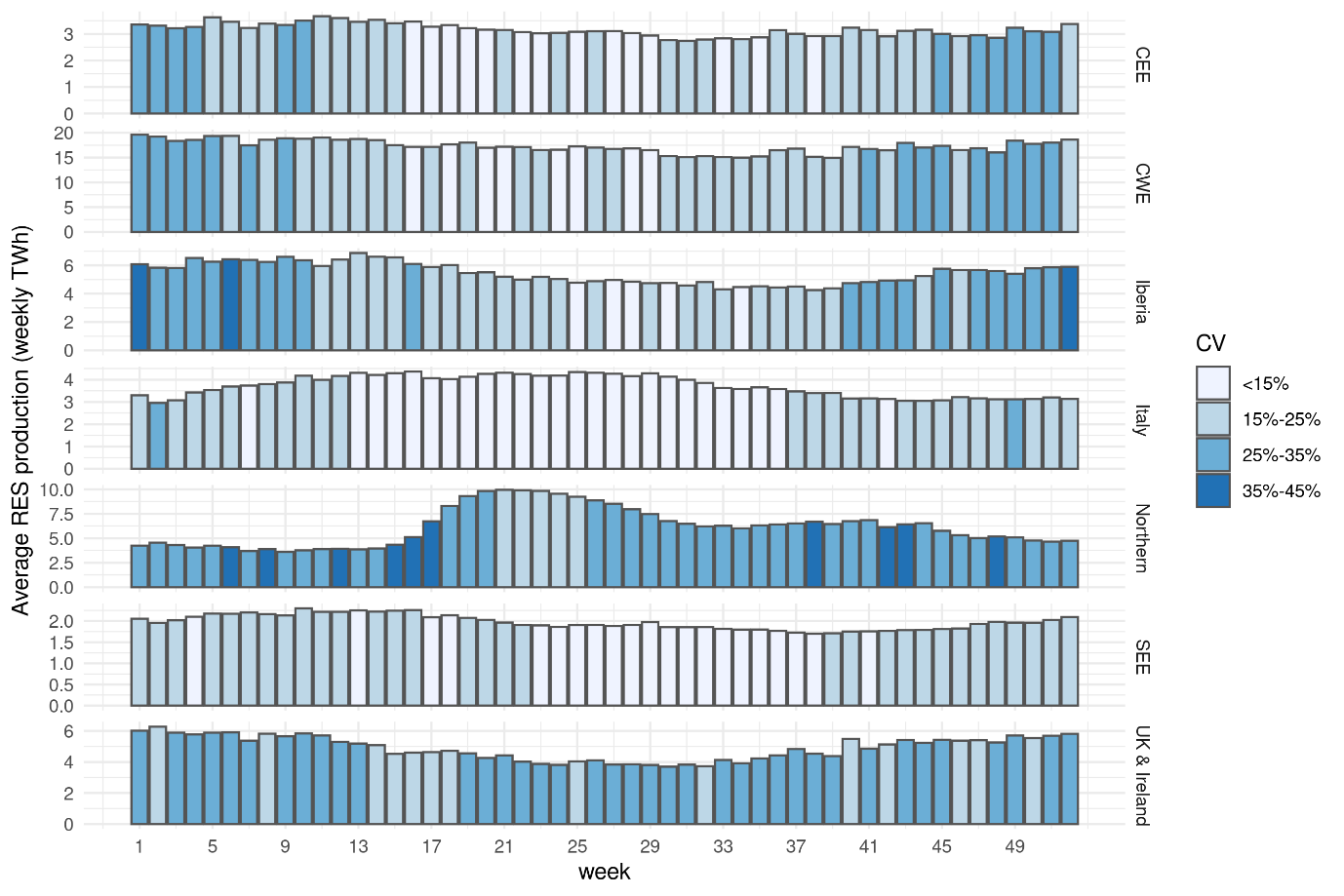


**Fig S2**. Weekly average production across all the regions for each RES in TWh. The colour represents the coefficient of variation for each week considering all the climate years


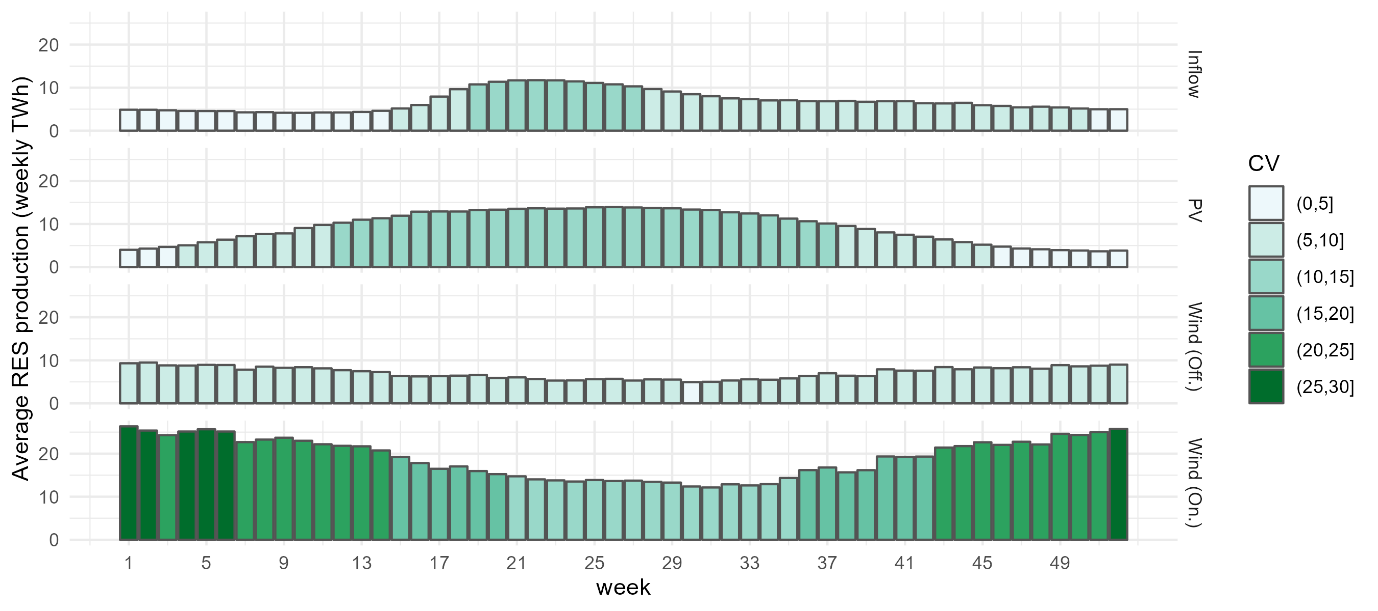


**Fig S3**. Annual variability of wind generation (onshore and offshore) as deviation from the long-term average (1982-2019) for all the regions.


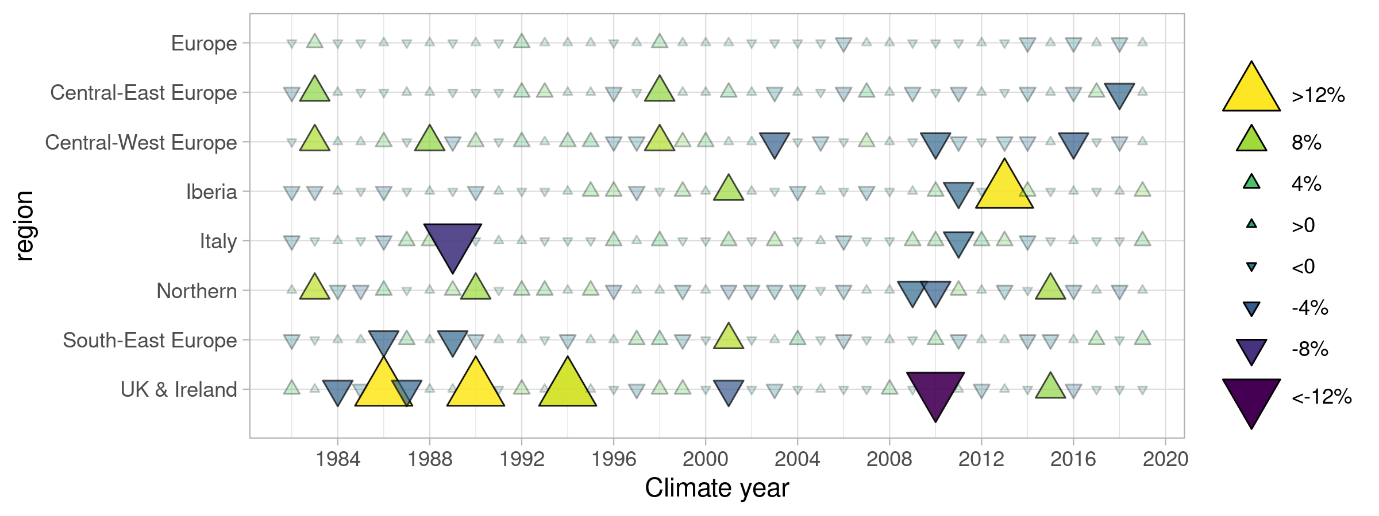


**Fig S4**. Annual variability of inflow as deviation from the long-term average (1982-2019) for all the region with hydropower capacity.


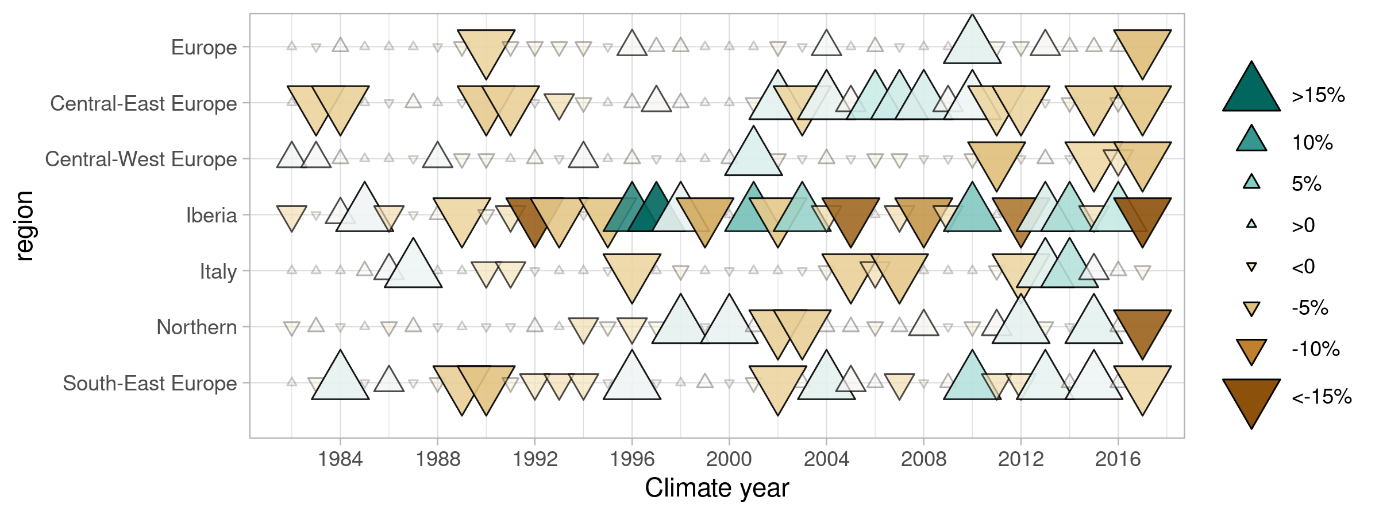


**Fig S5**. Annual variability of solar PV generation as deviation from the long-term average (1982-2019) for all the region*.*
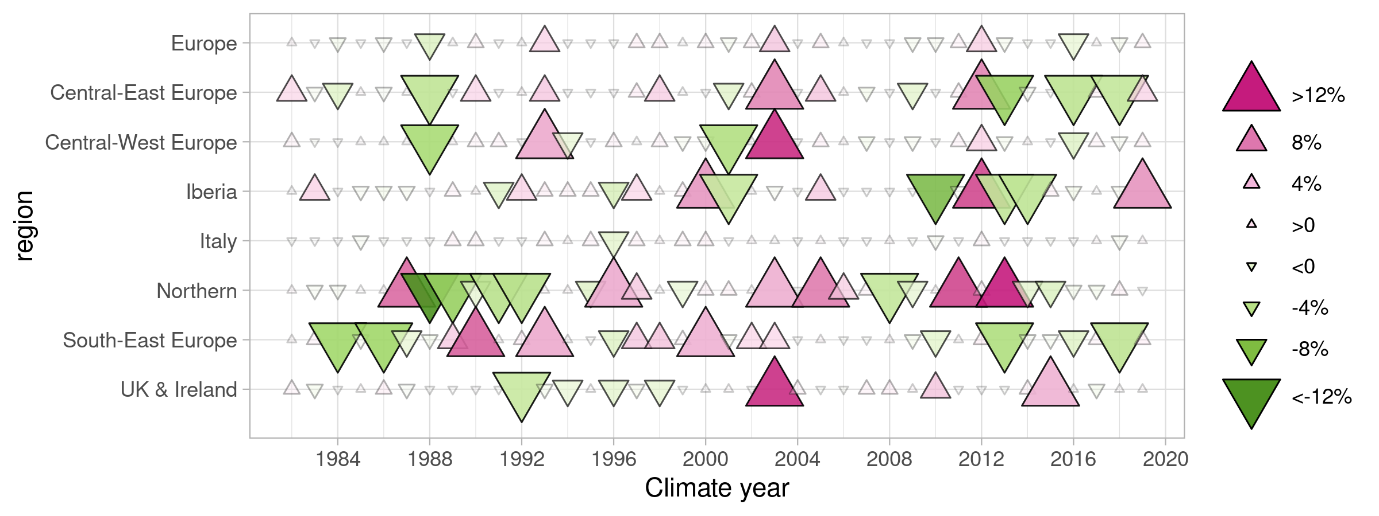

Supplement: S1 File — (DOCX) [file pone.0289705.s001.docx]
