# Supplementary material for: Climate variability on Fit for 55 European power systems
Source: PLoS One. 2023 Dec 20;18(12):e0289705. doi: 10.1371/journal.pone.0289705 (PMC10732370; doi:10.1371/journal.pone.0289705)
Supplement: Attachment — Submitted filename: response_to_reviewers.docx [file pone.0289705.s002.docx]

Dear editor,

Thank you for the effort that you put into reviewing our manuscript. We sincerely appreciate the invitation to address the concerns and resubmit our work to PLOS ONE. We have addressed the comments of the reviewers and believe this further improved the quality of our paper. In the following, we address the concerns in detail in commenting in a point-by-point manner.

Regarding the style, we would like also to clarify that:

1.	We have edited the document to match the PLOS ONE’s style requirements, changing references and cross-references

2.	We inserted in the manuscript the references to the two datasets available on Zenodo

3.	Figure 1 contains a map that we produced using R without any copyright

4.	Figure 4 has been published, as specified in the caption, on a journal with CC BY 4.0 license, therefore there is no need to ask for written permission because we follow the license requirements

We hope that you and the reviewer team like our revised paper. Please do not hesitate to contact us should any further questions arise.

Best regards,

Matteo De Felice, Derck Koolen, Konstantinos Kanellopoulos, Sebastian Busch, Andreas Zucker

**Response to reviewers**

We improved the writing and structure of the entire paper, incl. embedding the work in existing literature and a more clear explanation of our contribution. We have also checked the English language, updated structure of data, method and results and revised our discussion and conclusions. We improved the model description and added more comparison.

Here, we comment and discuss all the comments raised by the reviewers (in *italic*).

***Reviewer #1****: This paper covers an analysis of how climate year variability impacts power system optimization models. The researchers describe the variability of some model outcomes when the input data (renewable generation, and load) is based on different climatic years. It also describes how distinct regions correlate with each other in this regard. Finally, the impact of several known weather patterns on power system operations is described.*

*As a general remark, much greater attention should be paid to situating this work within the existing literature.*

> We have thoroughly reviewed the embedding of the work in existing literature. The updated structure more clearly addresses our contribution in relation to relevant literature (adding over 10 new references, and clarifying how this work is linked to the existing literature and its novelty).

*Note: page numbers of article start from 7, not 1.*

> We have checked and the page numbering is now ok.

*Major comments:*

*1. Overall it seems like the paper was written somewhat hastily, without sufficient proofreading. Please improve.*

> We have carefully revised the entire paper, improved the structure and rewritten large parts. The paper now has also been proofread by an English language editor.

*2. Abstract presents only motivation and methodology, please also present major findings and key messages or at the very least suggestions for the possible impact or use of this work and why (as you do on p.20 – 21).*

> The abstract has been entirely rewritten, also aiming to highlight the importance of our contribution and its relevance for wider readership of the journal.

*3. The description of the contribution of this work could also be made more clear in the beginning of the paper.*

> We have restructured the introduction, more explicitly highlighting the contribution of our work in a clear manner.

*4. p.8, l41 - Collins et al 2018 did not investigate climate variability in a planning context! They use a dispatch (= operational model).*

> Thank you for spotting this mistake. We removed that reference.

*1. Please compare your results to those of Collins et al 2018.*

> We compare now our findings with the results from Collins et al. 2018 (lines 196-203)

*5. More generally, the insights gained from the analysis of weather patterns are not distinguished from the existing literature: What are the lessons learned here, that were not yet found in other works?*

> We believe the article now better explains the novelty of our approach, stating for example (lines 259-260) that the used patterns (from Grams et al., 2017) are used for the first time in combination with a power system model, allowing us to associate them with cross-border exchanges or other energy variables (lines 371-373). Note that we have rewritten the entire discussion section, and believe the new structure in general better allows the reader to understand the relevance of the insights.

*6. p. 5, performance metrics*

*1. Please compare your performance metrics to those of Collins et al 2018.*

> The objective (and the scope) of our work is rather different from Collins et al. 2018, this is the reason why the metrics we use are different and difficult to compare with Collins’ work. The latter is focused on comparing current system (2015) with different scenarios (EU ref and ENTSO-E TYNDP four visions), using model-derived statistics (e.g., received prices) that can be compared (future vs baseline). Instead, our work tries to give some insight into the role of variability in a central policy scenario for EU power systems without any comparison with a baseline. We believe these differences justify the choice of the performance metrics.

We do agree that, given that both articles deal with the role of inter-annual variability, some discussion might help the reader to understand the link between the two articles. We therefore further clarified in line 161 the difference between both articles.

*2. Peak load is very uninformative for adequacy / reliability purposes. Peak net load (load – RES generation) or peak load net of power injection (load – generation + storage + demand response dispatch) would be more informative.*

> We agree with this comment, we changed from “peak load” to “peak net demand”

*7. Could you comment on how this variability impacts member states ability to reach targets?*

> Future EU power systems will need more flexibility to accommodate the variability in supply/demand and the link with weather conditions. Assessing this variability is crucial to include it in the energy modelling needed to support of the design of the future trajectories of all the power systems in EU. As stated in the recent regulation^[1]^ on the redesign of EU electricity markets: “Regulatory authorities should periodically assess the need for flexibility in the electricity system based on the input of transmission and distribution system operators” and also “On this basis, Member States should define a national objective for non-fossil flexibility such as demand side response and storage which should also be reflected in their integrated national energy and climate plans.” To clarify the relation with our article, we added in the paper a paragraph (from line 375) explaining the importance of our findings in light of EU policy making, including the aforementioned EU regulation.

*8. Model description is lacking. Please provide a brief but clear description of the model that is being used: temporal resolution, power flow approximation, technologies included, ..*

> We added a new section on Data & Models, providing a description of the model and referring the reader to the ref (De Felice, 2022; Sakellaris et al., 2018; European Commission, 2019b; Artelys, 2020) for a complete and in-depth discussion.

*9. Datasets can be described better. Data quality is of utmost importance for this type of analysis, yet no information is given on how the climatic variability has been correctly integrated in the datasets that have been used. I suggest to create an extra section in the paper that focuses on Data and Model description.*

> We have added a description of the data in the new Data & Models section and added an analysis on the variability of the used inputs as supplementary materials.

*1. p.8, Results – this includes description of the data and performance metrics, which should be in a separate Methodology section. Similarly some sections of the Introduction section could be moved there.*

> Please note again that we have revised and restructured the entire paper. We have moved all the information about the modelling and the used data in a new section, Data & Models. This section contains a description of the used data and the modelling framework.

*Minor comments:*

*1. p.7, l24-25 – how much of that capacity is variable RES (as opposed to e.g. biomass)?*

> We have taken the occasion to update the numbers using the more recent report (IEA, 2022) and we specified how much of the total RES capacity is from wind/solar.

*2. p.8 l.77 – please mention that the data is open source here (if I understood correctly).*

> We have specified that all the data used is open source (in line 129).

*3. The introduction states that the work covers two different aspects: 1) the co-variability among the European countries and 2) the identification of the predominant weather-based patterns. But then later in the paper, it is mentioned that the weather patterns have been taken from another manuscript, and this paper merely describes their influence on power system operation*

> We agree with the reviewer that the term ‘identification’ may be perceived as confusing. We now further explain in the article that we have used the pattern identified by Grams et al. (2018), analysing their impact on power systems operations.

*4. Could you elaborate on the parameters that you have chosen, why these and not others? E.g. curtailment, load shedding, line usage rates, ..*

The used power system models can reproduce the hourly operations of national power systems and indeed it generates hundreds of different variables. In the article, we focused on the variables the most describe the flows of electricity and the generation from renewables sources. For the sake of conciseness, we limited our analysis to a few indicators, but the model outputs we shared contain all the other indicators.

*Formatting:*

*1. Paragraphs are very short, sometimes just a single sentence. This makes for unnecessarily difficult reading.*

*2. Some very grammatically incorrect sentences e.g. “The change of CO2 intensity shows that during with the patterns AT, ScTr, Ar and EuBL the low-carbon generation across Europe seems following the same trend:” Please have a native English speaker check the paper.*

> We have carefully revised the document with the support of a professional editor.

***Reviewer #2****: Althouhg I find the topic interesting I think that given taht it seems you are referring to a long time span of years, just a descriptive statistics is not enough to describe the dataset. I would need to see the time evolution plots of the variables under study, so as to see at least if these series are stationary or not. Additionally you mention that the data is available but (maybe it was my fault) but I couldn't find it.*

*At least a descriptive analysis but taking into account that the data is evolving over time is needed for the paper being published.*

> We added a description of the used data in the new Data & Models section, including all relevant references. In addition, we have included additional information on data variability in the Supplementary Materials. We added the links to the input and output data shared with open license.

1. https://eur-lex.europa.eu/legal-content/EN/TXT/?uri=CELEX%3A52023PC0148&qid=1679410882233
